# Sangyod Rice Extract Attenuates Vascular Inflammation and Injury in a Rat Model of Diabetes by Modulating the Akt/MAPK Signaling Pathway

**DOI:** 10.1155/adpp/1169062

**Published:** 2025-04-17

**Authors:** Wanwipha Woonnoi, Kornsuda Thipart, Wanthanee Hanchang, Jirawat Saetan, Supita Tanasawet, Furoida Moolsup, Wiwit Suttithumsatid, Tulaporn Wongtawatchai, Wanida Sukketsiri

**Affiliations:** ^1^Division of Health and Applied Sciences, Faculty of Science, Prince of Songkla University, Hat Yai, Songkhla 90110, Thailand; ^2^Department of Physiology, Faculty of Medical Science, Naresuan University, Muang, Phitsanulok 65000, Thailand; ^3^Laboratory Animal Service Center, Faculty of Science, Prince of Songkla University, Songkhla 90110, Thailand; ^4^Department of Pharmacognosy and Pharmaceutical Botany, Faculty of Pharmaceutical Sciences, Prince of Songkla University, Songkhla 90110, Thailand

**Keywords:** endoplasmic reticulum stress, nuclear factor-kappa B, oxidative stress, proinflammatory cytokines, vascular complications

## Abstract

Our previous study has shown the beneficial effect of the ethanolic extract of Sangyod rice (SE) on lipid accumulation and insulin resistance. However, its effect on vascular inflammation has yet to be explored. The current study aimed to investigate the anti-inflammatory effects of SE in both in vitro and in vivo models, specifically examining its impact on LPS-induced inflammation in RAW264.7 cells and evaluating its efficacy in an animal model of diabetes mellitus induced by a high-fat diet combined with a low-dose streptozotocin. In the in vitro experiments, SE treatment effectively suppressed the LPS-induced activation of key signaling pathways, including Akt, ERK1/2, p38 MAPK, and NF-κB, which are known to play pivotal roles in the inflammatory response. SE was also found to reduce oxidative stress and the production of inflammatory markers in the LPS-stimulated RAW264.7 cells. In the in vivo experiments, the administration of SE (500 mg/kg BW) and metformin (200 mg/kg BW) to high-fat diet/streptozotocin-induced diabetic rats effectively improved dyslipidemia, as evidenced by reductions in serum total cholesterol, LDL-cholesterol, and triglycerides compared to the untreated diabetic control group. Importantly, SE ameliorated the damage to the vascular endothelium and elastic fibers by downregulating the expression of proinflammatory cytokines and oxidative stress markers. Additionally, SE administration attenuated the upregulation of key markers associated with ER stress-mediated apoptotic pathways, with effects comparable to those observed in diabetic rats treated with the standard antidiabetic drug metformin. These findings suggest that SE possesses both anti-inflammatory and vascular protective properties, evident in both in vitro and in vivo studies.

## 1. Introduction

Metabolic disorders, such as insulin resistance, visceral obesity, dyslipidemia, hyperglycemia, and hypertension, are major risk factors for the development of T2DM [1, 2]. Several evidences have shown that metabolic alterations can increase the risk of microvascular and macrovascular complications, including vascular endothelial cell injury and dysfunction. This can lead to the development of atherosclerosis, one of the diseases with the highest incidence rate and the leading cause of mortality among obese and diabetic patients worldwide [3, 4].

Excessive consumption of carbohydrates and fats can disrupt the balance in carbohydrate and fatty acid metabolism, which is a significant risk factor for metabolic syndrome. This imbalance can stimulate an inflammatory response attributed to the production of reactive oxygen species (ROS), NO, and/or ER stress. These factors play a crucial role in mediating the activation of several pathways involved in the pathogenesis of diabetes-related vascular injury and dysfunction [5–7]. The NF-κB is an important transcription factor related to the production of inflammatory cytokines and chemokines such as MCP-1, IL-1β, IL-6, TNF-*α*, and MMP enzymes that regulate chronic inflammatory diseases and vascular complications, leading to vascular endothelial cell dysfunction [8, 9]. Increasing evidence suggests that the inflammatory NF-κB signaling pathway can interact with various pathways, including MAPK. This involvement includes the ERK, JNK, and the p38, which control numerous processes associated with inflammatory cell responses implicated in the progression of T2DM-induced cardiovascular complications [10, 11]. The activated Akt also plays an important role in regulating a wide range of cellular processes, including differentiation, proliferation, migration, and apoptosis. Furthermore, the activation of ER stress has been related to the alteration of both structure and function of vascular endothelial cells, contributing to the progression of atherosclerosis [12, 13].

Rice (*Oryza sativa*) serves as the primary staple crop across Asia, including Thailand. Sangyod rice is classified as a geographic indication, indicating its unique characteristics tied to specific regions. It is a special variety of rice having a red-pericarp pigmentation, primarily cultivated in the Phatthalung province of southern Thailand [14]. As people become more health-conscious, they are increasingly consuming unpolished rice, especially red and dark-purple rice. These types of rice contain a rich phytochemical composition, including anthocyanins, procyanidins, phenolic acids, *γ*-aminobutyric acid, and gamma-oryzanol. These compounds offer several health-promoting benefits, such as reducing blood cholesterol levels, lowering high blood pressure, improving glucose metabolism, and decreasing the risk of atherosclerotic diseases [15–17]. Sangyod rice has gained much attention due to its strong antioxidant, anti-inflammatory, anti-diabetic activities, and cholesterol-lowering effects as demonstrated in both in vivo and in vitro experiments [14, 18–20]. Interestingly, our previous study showed that Sangyod rice extract decreased glucose uptake, lipid accumulation, and adipogenesis in 3T3-L1 adipocytes [14]. Furthermore, Sangyod rice bran hydrolysates have been demonstrated to prevent hypertension-related conditions with endothelial dysfunction, vascular remodeling, and oxidative stress in L-NAME-induced hypertension in rat models [21, 22]. However, there is still limited information on the effect of Sangyod rice ethanolic extract (SE) on the regulation of inflammation and oxidative stress associated with vascular endothelial cell injury under metabolic alterations. Therefore, the primary objective of this study was to investigate the effects of the SE in LPS-induced RAW264.7 cells and in an animal model fed a high-fat diet with low-dose streptozotocin against vascular injury. Furthermore, the study aimed to elucidate the underlying molecular mechanisms responsible for the observed effects of SE.

## 2. Materials and Methods

### 2.1. Preparation of SE

Sangyod rice, obtained from a local farmer in Phatthalung Province, Thailand, was extracted according to the method previously described by Hanchang et al. [20]. In brief, 10 kg of Sangyod rice was macerated in 80% of aqueous ethanol at a ratio of 1:10 (w/v) for 72 h and then filtered using Whatman No. 1 filter paper. Rotary evaporation under reduced pressure at 50°C was used to concentrate the extract. Phytochemical analysis of the SE was conducted using liquid chromatography and tandem mass spectrometry (LC–MS/MS) in both negative and positive modes, as presented in our previous study [20].

### 2.2. Cell Culture and Cytotoxicity Test

The murine macrophage RAW264.7 cell line (TIB-71; ATCC) was cultured as previously described [23]. Cytotoxicity was used to assess cell viability using the MTT assay. The RAW264.7 cells (1 × 10^4^ cells per well) were plated in a 96-well plate, allowed to adhere overnight, and then treated with SE for 24, 48, and 72 h at 1–500 μg/mL. Cell viability was detected at a 570-nm wavelength using a microplate spectrophotometer (Bio-Tex Instruments, Vermont, USA).

### 2.3. ROS Production by DCFH-DA Assay and NO Level by Griess Reagent

ROS and NO levels were measured following the method previously reported by Sukketsiri et al. [23]. In brief, 50,000 cells/well of RAW264.7 cells were incubated overnight onto a 96-well plate. Subsequently, 1 μg/mL of LPS (Sigma-Aldrich, MO, USA) was exposed to the cells in combination with or without SE (50 and 100 μg/mL) for 24 h. Intracellular ROS production was measured using the DCFH-DA assay, and NO levels in the culture medium were determined using the Griess reagent.

### 2.4. Immunocytochemistry

Immunocytochemistry was employed to ascertain the translocation of NF-κB. After treating the RAW264.7 cells with 1 μg/mL of LPS in combination with or without SE (50 and 100 μg/mL) for 24 h, the immunocytochemistry protocol was executed following the methodology previously described by Moolsap et al. [24]. Upon stimulation, cells were fixed with 4% paraformaldehyde for 15 min and permeabilized with 0.1% Triton X-100 for 15 min. Non-specific binding was blocked with 1% bovine serum albumin for 1 h. Subsequently, the cells were incubated overnight at 4°C with a primary anti-NF-κB antibody (SC-8008, 1:200), followed by a 1-h incubation at room temperature with Alexa Fluor 568-conjugated secondary antibodies (Invitrogen Life Technologies, USA). Nuclei were counterstained with Hoechst 33,342 (1 μg/mL; Sigma-Aldrich, MO, USA) and visualized with fluorescence microscopy (Olympus IX73, Japan).

### 2.5. Animal Model and Experimental Design

Rats (Sprague-Dawley; 5-week-old male) were obtained from Nomura Siam International (Bangkok, Thailand). The rats were kept in the animal facility (Naresuan University, Phitsanulok, Thailand) under controlled environmental conditions, including a 12:12-h light: dark cycle with 22 ± 1°C. The animals had ad libitum food and fluid access throughout the study period. All procedures involving the animals were carried out regarding the ethical approval (NU-AE650710; the Animal Ethics Committee of Naresuan University). The protocol for inducing diabetes in rats, along with all blood samples and vessels used in this study, was derived from our previous research [20]. The study utilized a rat model of T2DM induced by a high-fat diet and streptozotocin. Sprague-Dawley rats were fed a high-fat diet (60.3% kcal fat, 21.4% kcal carbohydrate, 18.3% kcal protein, Envigo, Indianapolis, USA) for 3 weeks. After the diet, rats in the high-fat diet group received an intraperitoneal injection of streptozotocin (35 mg/kg; Merck Millipore, MA, USA), while the normal control group, fed a normal diet, received a citrate buffer injection. Rats with fasting blood glucose levels ≥ 200 mg/dL 5 days after streptozotocin induction were considered T2DM models and selected for subsequent experiments and were randomly divided into five experimental groups with seven animals per group: (I) Normal control group (Control): normal rats received 50% polyethylene glycol; (II) diabetic group (DM): T2DM induced by a high-fat diet and streptozotocin received 50% polyethylene glycol; (III) diabetic + metformin group (DM + M200): T2DM induced by a high-fat diet and streptozotocin received 200 mg/kg BW of metformin, a standard antidiabetic medication; (IV) diabetic + SE (250 mg/kg) group (DM + SE250): T2DM induced by a high-fat diet and streptozotocin received 250 mg/kg BW of SE; and (V) diabetic + SE (500 mg/kg) group (DM + SE500): T2DM induced by a high-fat diet and streptozotocin received 500 mg/kg BW of SE. At the end of the study (30 days), the rats were fasted overnight before receiving intraperitoneally thiopental sodium (100 mg/kg). Blood samples and the aorta were then collected for further analysis. The aortic tissue was removed, cleaned with saline solution, and then weighed on an analytical balance.

### 2.6. Determination of Serum Lipid Profiles and Oxidative Stress Markers

The TC, LDL-C, TG, and HDL-C in serum were evaluated at N Health Laboratory (Bangkok, Thailand). The TP and TAS levels in serum were analyzed to determine the OSI in serum, as previously reported by Choosri et al. [25]. The TP level was determined using the FOX assay at 560 nm, with hydrogen peroxide serving as a standard solution. For TAS measurement, plasma was mixed with reagent 1 (acetate buffer, 0.4 mol/L, pH 5.8), followed by reagent 2 (ABTS^+^ in acetate buffer, 30 mmol/L, pH 3.6). The TAS level was then determined by measuring the absorbance at 660 nm, using Trolox as a standard. The OSI was calculated using the formula OSI = (TP/TAS) × 100.

### 2.7. Histological Examination

After fixation of aortic tissue samples in 4% paraformaldehyde solution, the fixed samples underwent dehydration, clearing, and embedding in paraffin blocks. The embedded aortic samples were serially sectioned into 5-μm thick sections using a microtome (Leica Microsystems, Germany). Following sectioning, the tissue sections were hydrated in a descending series of alcohol concentrations (100%, 95%, and 75%). The hydrated tissue sections were stained with Elastica van Gieson (Sigma-Aldrich, MO, USA) and Masson's trichrome (Sigma-Aldrich, MO, USA) staining to evaluate elastic fiber content and fibrosis, respectively. After staining, the tissue sections were examined under a DP73 light microscope (Olympus, Japan) with the cellSens imaging software to assess the histopathological changes and the distribution of elastic fibers and fibrosis within the aortic tissue samples.

### 2.8. mRNA Expression by Real-Time RT-PCR

The homogenized aortic tissue samples were used to extract total RNA using TRIzol reagent (Invitrogen Life Technologies, CA, USA). Next, the DNase-treated RNA was used to reverse transcribe the RNA into cDNA using a Superscript VILO cDNA synthesis kit (Invitrogen Life Technologies) at 42°C for 1 h. The resulting cDNA was utilized for gene expression analysis by reverse transcription quantitative PCR (RT-qPCR), following the procedure and primer sequences previously described by Chusongdam et al. [26].

### 2.9. Protein Expression by Western Blot

After treating RAW264.7 cells with LPS (1 μg/mL) in combination with or without SE (at concentrations of 50 and 100 μg/mL) for 24 h, each cell sample was lysed in RIPA buffer containing a protease inhibitor. Additionally, homogenized aortic tissue samples were prepared in a similar manner. Then, the Bradford protein assay (Bio-Rad, California, USA) was carried out to measure protein quantitation in each sample. Western blot procedures were conducted as previously reported by Woonnoi et al. [14]. Briefly, 75 μg of protein samples were separated using sodium dodecyl-sulfate polyacrylamide gel electrophoresis and subsequently transferred onto a polyvinylidene difluoride membrane. The membrane was blocked with 5% skim milk for 2 h to prevent non-specific binding and incubated overnight at 4°C with specific primary antibodies against the following proteins: Akt (SC-81434, 1:1000), pAkt (SC-514032, 1:1000), ERK1/2 (ab36991, 1:1000), pERK1/2 (ab50011, 1:1000), p38 (ab31828, 1:1000), p-p38 (ab4822, 1:1000), iNOS (SC-7271, 1:1000), BAX (#2772, 1:1000), Bcl-2 (#3498, 1:1000), cleaved-caspase 3 (ab32042, 1:1000), pro-caspase 3 (ab184787, 1:1000), and β-actin (1:1000; Invitrogen Life Technologies, USA). Thereafter, a horseradish peroxidase-conjugated secondary antibody (1:15,000; Invitrogen Life Technologies, USA) was incubated for 2 h at room temperature. All protein band intensities were normalized to β-actin.

### 2.10. Statistical Analysis

The data are expressed as the mean ± standard error of the mean (SEM). A one-way analysis of variance (ANOVA) was performed, and subsequently Duncan's *post hoc* test was used to assess the specific differences among groups. A *p* value less than 0.05 was considered statistically significant.

## 3. Results

### 3.1. Cytotoxicity and SE Inhibit the ROS and NO Levels in LPS-Induced RAW264.7 Cells

**Figure 1:**
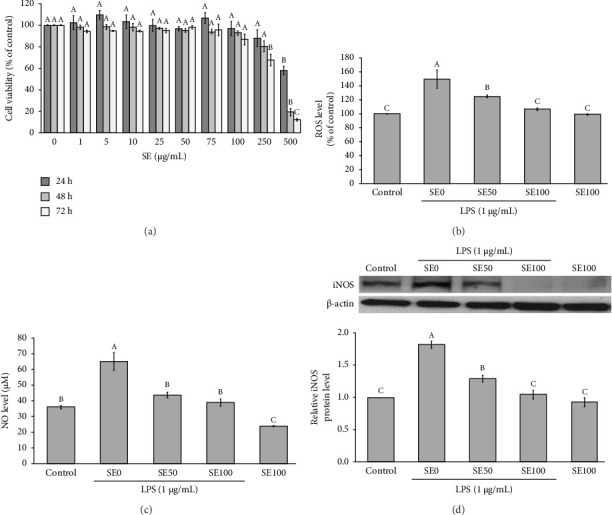
Effects of SE on reactive oxygen species (ROS) and nitric oxide (NO) levels in lipopolysaccharide (LPS)-induced RAW264.7 macrophage cells. (a) Cytotoxicity of SE, (b) ROS level, (c) NO level, and (d) Western blot analysis of inducible nitric oxide synthase (iNOS) protein expression. All data are expressed as mean ± SEM, with *n* = 4 per group. Different uppercase letters denote statistical significance at *p* ≤ 0.05. SE0 = 0 μg/mL of SE and 1 μg/mL of LPS, SE50 = 50 μg/mL of SE and 1 μg/mL of LPS, SE100 = 100 μg/mL of SE and 1 μg/mL of LPS.

The cytotoxicity of SE on RAW264.7 cells was initially assessed using the MTT assay.  Figure 1(a) illustrates that the low concentrations (1–100 μg/mL) of SE did not affect the RAW264.7 cells viability at all time points of incubation (24, 48, and 72 h). However, the high concentrations of SE (250–500 μg/mL) notably decreased the RAW264.7 cell viability. Based on these findings, the non-toxic concentrations of SE at 50 and 100 μg/mL were chosen for subsequent experiments. Regarding ROS levels, exposure to LPS alone led to a notable increase in ROS levels, reaching approximately 149.88 ± 12.95% of the control in RAW264.7 cells. However, the treatment of LPS-induced RAW264.7 cells with 100 μg/mL of SE resulted in a significant reduction in ROS levels compared to the LPS alone group (SE0) (Figure 1(b)). Moreover, a significant increase in NO levels and iNOS protein expression was observed in the group treated with LPS alone. Interestingly, both NO levels and iNOS expression markedly decreased after LPS-stimulated RAW264.7 cells were treated with 50 μg/mL and 100 μg/mL of SE (Figures 1(c) and 1(d)). These results indicate that SE exhibits anti-inflammatory properties against LPS-induced macrophages by reducing inflammatory mediators such as ROS and NO.

### 3.2. SE Inhibits the Activation of Akt, ERK1/2, and p38 MAPK Signaling in LPS-Induced RAW264.7 Cells

**Figure 2:**
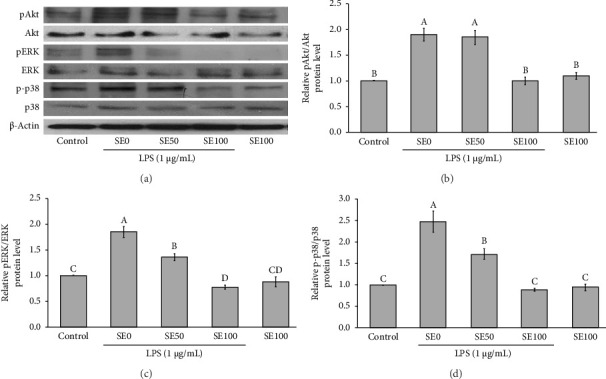
SE inhibited the activation of Akt, ERK1/2, and p38MAPK signaling pathways in LPS-induced RAW264.7 cells. (a) Western blot analysis of Akt, ERK1/2, and p38 MAPK, (b) relative expression of pAkt/Akt, (c) relative expression of pERK/ERK, and (d) relative expression of p-p38/p38. All data are expressed as mean ± SEM, with *n* = 4 per group. Different uppercase letters denote statistical significance at *p* ≤ 0.05. SE0 = 0 μg/mL of SE and 1 μg/mL of LPS, SE50 = 50 μg/mL of SE and 1 μg/mL of LPS, and SE100 = 100 μg/mL of SE and 1 μg/mL of LPS.

The results showed that upon the stimulation of RAW264.7 cells with LPS, the phosphorylation of key signaling proteins, including Akt, ERK1/2, and p38 MAPK, was significantly enhanced without affecting the total protein levels after 24 h of treatment (Figures 2(a), 2(b), 2(c), 2(d)). Importantly, the addition of SE, particularly at its highest concentration, inhibited the activation of Akt, ERK1/2, and p38 MAPK in the LPS-induced RAW264.7 cells (Figures 2(a), 2(b), 2(c), 2(d)). This finding confirms the anti-inflammatory effects of SE, which were regulated by the suppression of the Akt and MAPK signaling pathways.

### 3.3. SE Hinders the Translocation of NF-κB in LPS-Induced RAW264.7 Cells

**Figure 3:**
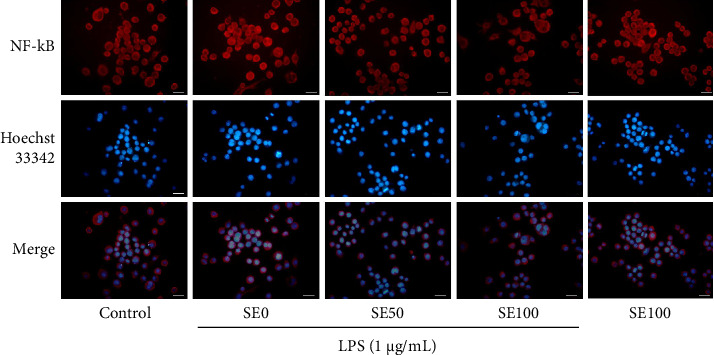
SE inhibits p65 NF-κB translocation in LPS-induced RAW264.7 cells. The cells were stained with NF-κB antibody and Hoechst 33,342 and then visualized using fluorescence microscopy (20 μm scale bar). SE0 = 0 μg/mL of SE and 1 μg/mL of LPS, SE50 = 50 μg/mL of SE and 1 μg/mL of LPS, and SE100 = 100 μg/mL of SE and 1 μg/mL of LPS.

As depicted in Figure 3, the nuclear intensity of p65 NF-κB was upregulated in the group treated with LPS alone. Notably, after LPS-induced RAW264.7 cells were exposed to SE, the nuclear translocation of p65 NF-κB was subsequently decreased. These findings highlight the anti-inflammatory properties of SE through the suppression of the NF-κB inflammatory cascade.

### 3.4. Effects of SE on Aorta Weight, Serum Lipid Profiles, and Oxidative Stress Index

The study investigated the effects of SE on blood lipid levels and oxidative stress in diabetic rats. The results showed that, compared to the control group, serum levels of TC, TG, and LDL-C were significantly higher in the DM group (Table 1). However, following treatment with SE, there was a notable decrease in serum TC, LDL-C, and TG levels, particularly at the highest dose compared to the DM group. Additionally, SE decreased the serum lipid profile (TC, TG, and LDL-C) in diabetic rats similar to the standard drug metformin. However, the concentration of HDL-C did not differ significantly between the experimental groups and the control group (Table 1). For serum OSI, levels of TP and OSI in rat serum were notably elevated in the DM group compared to those in the control group. Following SE treatment, there was a significant reduction in TP and OSI levels, particularly at the highest dose of SE. This effect was similar to that observed with the standard drug metformin (Table 1). These results implied that treatment with SE could reduce serum lipid levels and oxidative stress in diabetic rats.

### 3.5. SE Ameliorated Pathological Changes in Aorta of DM Rats

**Figure 4:**
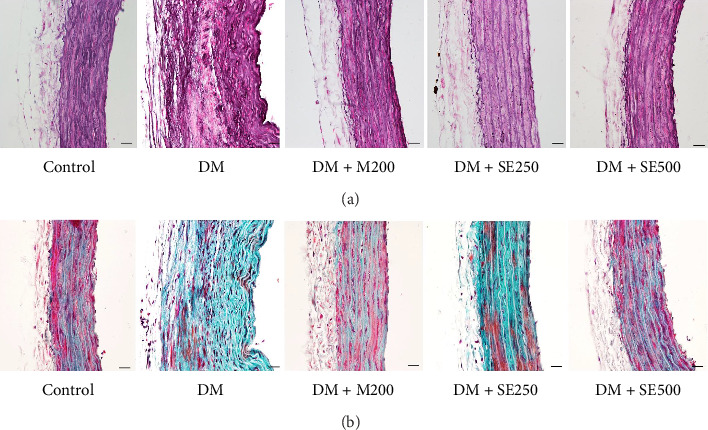
SE ameliorated pathological changes in aorta of DM rats. The aorta histological sections (40x) stained with (a) elastic fiber and (b) Masson's trichrome. Control: normal control rats received a normal diet and 50% polyethylene glycol, DM: diabetic rats received a HFD and 50% polyethylene glycol, DM + M200: diabetic rats received HFD and 200 mg/kg BW of metformin, a standard antidiabetic medication, DM + SE250: diabetic rats received HFD and 250 mg/kg BW of SE, and DM + SE500: diabetic rats received HFD and 500 mg/kg BW of SE.

As depicted in Figures 4(a) and 4(b), the vascular endothelium and elastic fiber structure in the control group appeared clear and intact, with smooth muscle cells arranged neatly without observable pathological changes. In contrast, the DM group exhibited disrupted vascular endothelium and damaged elastic fibers. The tunica intima showed significant thickening with evident hyperplasia and a notable accumulation of foam-like smooth muscle cells. Furthermore, both metformin and SE (500 mg/kg BW) treatments demonstrated a significant protective effect against vascular lesions, reducing the extent of elastic fiber damage and the accumulation of foam-like smooth muscle cells.

### 3.6. SE Suppresses the mRNA Expression of Proinflammatory Cytokine in Aorta Tissues of DM Rats

**Figure 5:**
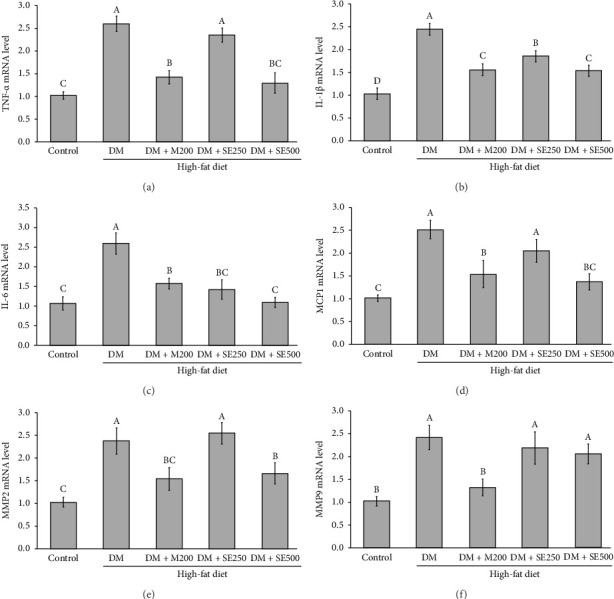
Effects of SE on the expression of key proinflammatory cytokine genes in a diabetic rat model. (a) *TNF-α* gene expression, (b) *IL-1β* gene expression, (c) *IL-6* gene expression, (d) *MCP-1* gene expression, (e) *MMP2* gene expression, and (f) *MMP9* gene expression. All values are presented as mean ± SEM, with *n* = 6 per group. Different uppercase letters denote statistical significance at *p* ≤ 0.05. Control: normal control rats received a normal diet and 50% polyethylene glycol, DM: diabetic rats received a HFD and 50% polyethylene glycol, DM + M200: diabetic rats received HFD and 200 mg/kg BW of metformin, a standard antidiabetic medication, DM + SE250: diabetic rats received HFD and 250 mg/kg BW of SE, and DM + SE500: diabetic rats received HFD and 500 mg/kg BW of SE.

Figure 5 illustrates the assessment of proinflammatory cytokine gene expression in the rat abdominal aorta. The expression of proinflammatory cytokines TNF-α, IL-1β, IL-6, MCP-1, MMP-2, and MMP-9 in the DM group was significantly upregulated compared to that in the control group (Figures 5(a), 5(b), 5(c), 5(d), 5(e), 5(f)). The upregulation of TNF-α, IL-1β, IL-6, MCP-1, and MMP-2 mRNA expression was significantly suppressed at the highest dose of SE treatment, while MMP-9 expression remained unaffected. In contrast, metformin treatment significantly downregulated the expression of all proinflammatory cytokine genes (Figures 5(a), 5(b), 5(c), 5(d), 5(e), 5(f)).

### 3.7. SE Inhibited the ER Stress-Induced Apoptosis in Aorta Tissues of DM Rats

**Figure 6:**
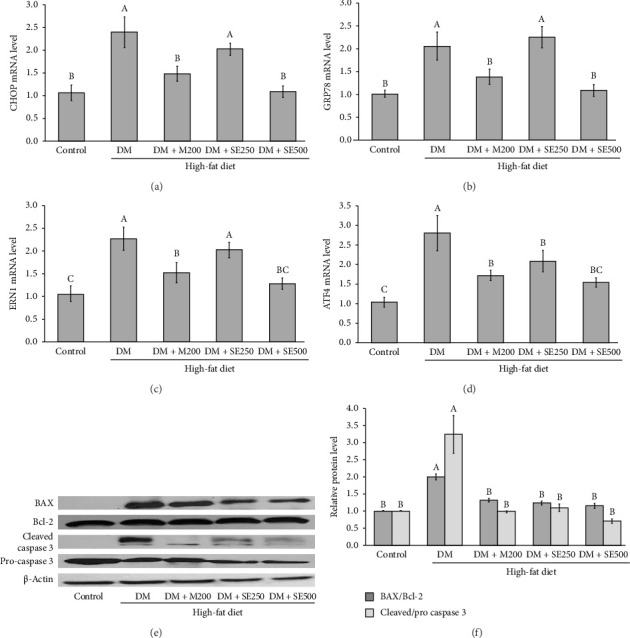
The influence of SE on the expression of ER stress markers and apoptotic protein in a diabetic rat model. (a) *CHOP* gene expression, (b) *GRP78* gene expression, (c) *ERN1* gene expression, (d) *ATF4* gene expression, (e) Western blot analysis of Bax, Bcl-2, procaspase 3, and cleave-caspase 3 proteins, and (f) relative expression of Bax/Bcl-2 and cleave caspase 3/pro-caspase 3. All values are presented as mean ± SEM, with *n* = 6 per group. Different uppercase letters denote statistical significance at *p* ≤ 0.05. Control: normal control rats received a normal diet and 50% polyethylene glycol, DM: diabetic rats received a HFD and 50% polyethylene glycol, DM + M200: diabetic rats received HFD and 200 mg/kg BW of metformin, a standard antidiabetic medication, DM + SE250: diabetic rats received HFD and 250 mg/kg BW of SE, and DM + SE500: diabetic rats received HFD and 500 mg/kg BW of SE.

ER stress marker genes, such as CHOP, GRP78, ATF4, and ERN1, were significantly upregulated in the DM group compared to those in the control group (Figures 6(a), 6(b), 6(c), 6(d)). In both metformin and SE treatments, the gene expressions of CHOP, GRP78, ATF4, and ERN1 were notably decreased (1.48 ± 0.17, 1.38 ± 0.16, 1.71 ± 0.13, and 1.51 ± 0.252 for DM + M200; 1.09 ± 0.13, 1.08 ± 0.14, 1.53 ± 0.11, and 1.27 ± 0.12 for DM + SE500) compared to those in the DM group (Figures 6(a), 6(b), 6(c), 6(d)). The effect observed with SE was similar to that observed with metformin. The study also examined the effects of SE and metformin on the expression of apoptosis-related proteins in the aortic tissue of diabetic rats. The results showed that the Bax/Bcl-2 ratio and the cleaved-caspase-3/pro-caspase-3 ratio were significantly downregulated in the metformin and SE treatment groups compared to those in the DM group, as depicted in Figures 6(e) and 6(f). These findings suggest that SE treatment may alleviate vascular injury by inhibiting ER stress-induced apoptosis in the aortic tissue of diabetic rats.

## 4. Discussion

Vascular complications are prevalent among diabetic patients. Metabolic abnormalities, including hyperglycemia, insulin resistance, and hyperlipidemia, contribute to vascular injury by causing endothelial dysfunction and increased oxidative stress, thereby exacerbating atherosclerosis [5, 27]. In particular, excessive consumption of a high-fat diet leading to obesity and metabolic disorders has also been considered a risk factor for vascular alterations in diabetic patients. The accumulation and oxidation of lipids can activate an inflammatory process, which in turn activates ROS production and the ER stress, causing structural alterations in the vasculature and subsequently endothelial dysfunction. This dysfunction is accompanied by foam cell formation, arterial wall thickening, plaque rupture, and occluding thrombus. These contribute to the progression of the initial stages of atherosclerosis in diabetic patients and other cardiovascular complications [5, 6]. In this study, we demonstrated the anti-inflammatory properties of SE in RAW264.7 cells as well as highlighted the effect of SE on vascular injury in a diabetic rat model induced by high-fat diet/streptozotocin. We found that the diabetic rat model induced by high-fat diet/streptozotocin exhibited significantly increased levels of lipid profiles (TC, TG, and LDL-C), TP, and OSI in serum. Moreover, histological changes in the rat's abdominal aortas showed thickened tunica intima and foam-like smooth muscle cell formation in high-fat diet/streptozotocin-induced DM rats, implying vascular endothelial cell disruption. Our findings are consistent with previous studies indicating that a high-fat diet combined with low-dose streptozotocin results in elevated serum TG, TC, and oxidative stress, as well as reduced antioxidant defenses in diabetic animal models [28–30]. This suggests that a hyperlipidemic condition and free radical generation are commonly associated with diabetes, increasing the risk of cardiovascular diseases [29, 30]. Interestingly, treatment with SE and metformin as antidiabetic therapies resulted in a significant reduction in serum lipid profiles (TC, TG, and LDL-C), TP, and OSI. Additionally, these treatments alleviated the severity of vascular structural changes and elastic fiber damage compared to the DM model group, indicating the protective effects of SE against vascular injury by preventing the formation of foam-like smooth muscle cells in high-fat diet/streptozotocin-induced DM rats. Previous studies by Hanchang et al. [20] and Woonnoi et al. [14] have shown that SE contains bioactive compounds such as phenolic compounds, terpene glycosides, proanthocyanidins, and alkaloids. These bioactive ingredients may contribute to the protective effects of SE observed in this study. Consistent with our findings, several studies have demonstrated that phenolic and flavonoid compounds from natural sources exhibit anti-hyperlipidemic and antioxidant activities in rats fed a high-fat diet [31, 32]. SE has been shown to inhibit adipogenesis by reducing lipid accumulation and triglyceride levels in adipocytes, as well as a decreased lipid buildup in the liver tissue of DM rats [14, 20]. The beneficial effects of SE may be partly attributed to its phenolic constituents, which can protect against hypercholesterolemia by lowering serum TC, TG, and LDL-C levels.

Oxidative stress and chronic inflammation are closely linked to the development and progression of atherosclerosis, particularly in the context of metabolic disorders such as diabetes [33]. The upregulation of ROS can lead to auto-oxidation of proteins and nucleic acids through inflammatory pathways involving Akt, ERK, and p38 MAPKs [23, 24]. These pathways activate transcription factors like NF-κB, leading to an increased expression of proinflammatory mediators such as iNOS, COX-2, IL-6, IL-1β, and TNF-α. These mediators are closely linked to chronic conditions including diabetes, obesity, atherosclerosis, and cancer [20, 23, 34]. Thus, inhibiting signaling pathways and/or inflammatory mediators is an important focus for therapeutic alternative strategies in inflammatory diseases. Our findings indicate that SE exhibits antioxidant properties by suppressing ROS production and anti-inflammatory effects by inhibiting LPS-stimulated responses. This occurs through the suppression of Akt and MAPK (ERK1/2 and p38) signaling pathways as well as preventing NF-κB subunit translocation into the nucleus. Consequently, it inhibits iNOS protein expression and reduces NO production in RAW264.7 cells. Previous research has shown that SE contains a variety of biologically active polyphenolic compounds [14, 20], which possess several pharmacological activities. For instance, caffeic acid, a compound found in these extracts, exhibits both antioxidant and anti-inflammatory properties. This might possibly be due to the inhibition of NF-κB and MAPK cascades in response to proinflammatory stimuli such as LPS. Furthermore, caffeic acid has been found to suppress oxidative stress and ER stress in human endothelial cells [35, 36]. Our findings showed that the anti-inflammatory properties of SE observed in the in vitro study are consistent with those found in the high-fat diet/streptozotocin-induced diabetic rat model. This consistency between in vitro and in vivo findings supports the properties of SE across different experimental settings. Long-term exposure to high-fat diets or hyperglycemia can impair endothelial cell function via inflammatory cytokines such as TNF-α, IL-1β, IL-6, and MCP-1 [37, 38]. Moreover, hyperglycemia-induced oxidative stress plays a critical role in activating the expression of MMPs, particularly MMP-2 and MMP-9, which have been implicated in various vascular conditions associated with diabetes [39]. Our study found elevated mRNA expression levels for TNF-α and other cytokines/MMPs in diabetic rats' abdominal aortas. However, treatment with SE significantly suppressed the mRNA expression of TNF- *α*, IL-1β, IL-6, MCP-1, and MMP-2, similar to metformin treatment. These findings are similar to previous studies that have demonstrated the antihypertensive and antioxidant effects of Sangyod rice bran hydrolysates against high blood pressure, oxidative stress, and endothelial dysfunction in hypertensive rats [21, 22]. Therefore, these findings suggest that SE exhibits properties similar to metformin, including anti-inflammatory and antioxidant effects, potentially protecting against vascular injury by reducing proinflammatory cytokines and oxidative stress associated with the development of diabetes-related atherosclerosis.

Several risk factors, including metabolic disorders, dyslipidemia, hyperglycemia, hypertension, oxidative stress, and inflammatory responses, contribute to ER stress. These factors disrupt ER homeostasis and potentially lead to the progression of various cardiovascular diseases [13, 20, 40]. Our study demonstrated that high-fat diet/streptozotocin-induced diabetic rats exhibited a significant increase in the gene expression of ER stress markers, including CHOP, GRP78, ATF4, and ERN1, in their abdominal aortas. Furthermore, these conditions also enhanced apoptosis-related markers such as Bax/Bcl-2 and cleaved-caspase 3/pro-caspase 3 ratios. Consistent with previous studies [20, 41, 42], hyperglycemia and long-term high-fat diets activated ER stress by upregulating GRP78, CHOP, and ATF4. This activation led to chronic inflammation and endothelial cell apoptosis. The administration of SE and metformin significantly reduced the expression levels of ER stress markers, including CHOP, GRP78, ATF4, and ERN1, in the abdominal aortas of high-fat diet/streptozotocin-induced diabetic rat models. Both treatments effectively suppressed apoptosis by decreasing Bax/Bcl-2 and cleaved-caspase 3/pro-caspase 3 ratios. The ability of SE to modulate ER stress-related proteins and apoptotic pathways suggests that it may possess therapeutic properties useful in mitigating diabetic vascular complications, such as atherosclerosis. Our previous studies have found that SE contains ferulic acid and procyanidin B2 [14, 20]. Ferulic acid has been reported to exert beneficial effects on vascular function in heart diseases, particularly under conditions of severe ER stress in H9c2 cells and isolated ventricular myocytes from rat models [43]. Recently, procyanidin B2 has been reported to improve endothelial dysfunction by downregulating ER stress-related signaling molecules, including CHOP, GRP78, and ATF4 [44]. The observed reduction in ER stress marker genes and apoptotic proteins may be attributed to compounds such as ferulic acid and procyanidin B2 present in SE. This result confirms that the effect of SE in modulating both ER stress pathways and apoptotic mechanisms indicates its effectiveness in inhibiting diet-induced vascular injury by suppressing apoptosis triggered by ER stress.

## 5. Conclusion

**Figure 7:**
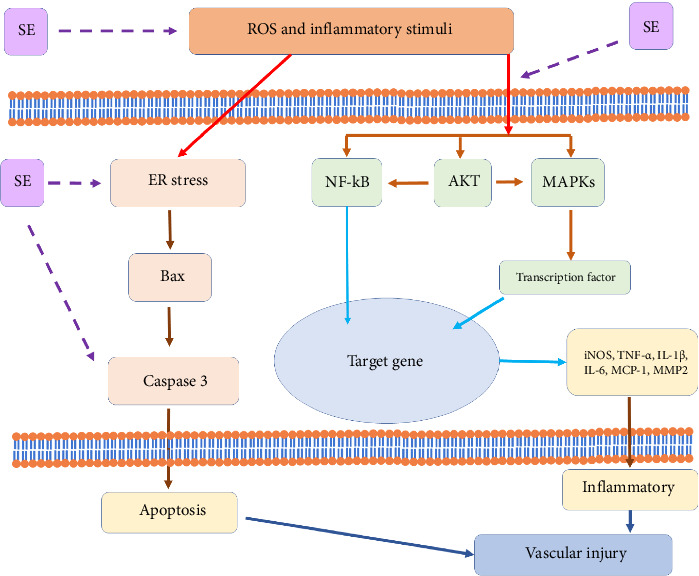
The mechanism of Sangyod rice extract modulating vascular injury.

The present findings suggest that SE exerts anti-inflammatory and anti-apoptotic effects against vascular injury in a rat model of high-fat diet and low-dose streptozotocin-induced diabetic vascular complications. The pharmacological actions of SE treatment are possibly attributed to its ability in reducing oxidative stress, inhibiting proinflammatory cytokines, and alleviating ER stress-induced apoptosis-related signaling molecules. In addition, SE significantly inhibited inflammatory mediator, possibly via downregulation of the expression of Akt/MAPK and NF-κB signaling pathways in LPS-stimulated inflammation in RAW264.7 cells (Figure 7). These findings suggest that SE may be a promising nutraceutical candidate for the prevention and management of vascular complications associated with diabetes. Further investigation into the underlying mechanisms, dose-dependent effects, and long-term efficacy of SE in preclinical and clinical settings would be valuable to establish its therapeutic potential in the context of diabetic vascular complications.

## Figures and Tables

**Table 1:** Effects of SE on aorta weight, serum lipid profiles, and oxidative stress index.

Parameters	Control	DM	DM + M200	DM + SE250	DM + SE500
Serum cholesterol (mg/dL)	50.29 ± 3.09^c^	72.86 ± 3.56^a^	51.29 ± 4.64^c^	62.00 ± 2.75^b^	43.14 ± 4.80^c^
Serum HDL (mg/dL)	19.43 ± 0.95^a^	18.29 ± 1.21^a^	18.00 ± 1.66^a^	18.57 ± 1.98^a^	21.29 ± 1.70^a^
Serum LDL (mg/dL)	4.86 ± 0.67^b^	16.57 ± 3.81^a^	5.71 ± 0.56^b^	13.29 ± 1.77^a^	7.43 ± 1.87^b^
Serum triglyceride (mg/dL)	73.40 ± 6.95^b^	120.86 ± 8.36^a^	47.14 ± 12.68^b^	97.57 ± 29.06^a^	80.43 ± 12.20^b^
Serum TP level (μmol H_2_O_2_/L)	10.33 ± 0.16^c^	29.39 ± 0.19^a^	10.01 ± 0.10^c^	21.18 ± 0.18^b^	10.00 ± 0.11^c^
Serum TAS level (mmol trolox equiv/L)	4.02 ± 0.01^c^	4.11 ± 0.02^b^	4.31 ± 0.02^a^	4.09 ± 0.1^b^	4.06 ± 0.03^bc^
Serum oxidative stress index (OSI)	0.26 ± 0.01^c^	0.72 ± 0.01^a^	0.23 ± 0.01^d^	0.52 ± 0.01^b^	0.25 ± 0.01^c^
Aorta weight (g)	0.22 ± 0.03^a^	0.28 ± 0.02^a^	0.26 ± 0.04^a^	0.25 ± 0.02^a^	0.24 ± 0.03^a^

*Note:* All values are presented as mean ± SEM (*n* = 7). Different lowercase letters denote statistical significance at *p* ≤ 0.05. Control: normal control rats received a normal diet and 50% polyethylene glycol, DM: diabetic rats received a HFD and 50% polyethylene glycol, DM + M200: diabetic rats received HFD and 200 mg/kg BW of metformin, a standard antidiabetic medication, DM + SE250: diabetic rats received HFD and 250 mg/kg BW of SE, and DM + SE500: diabetic rats received HFD and 500 mg/kg BW of SE.

## Data Availability

The data that support the findings of this study are available from the corresponding author upon reasonable request.

## Nomenclature

Akt: Protein kinase B
ATF4: Activating transcription factor 4
Bax: B-cell lymphoma protein 2-associated X
Bcl-2: B-cell lymphoma protein 2
CHOP: CCAAT-enhancer-binding protein homologous protein
COX-2: Cyclooxygenase 2
ER: Endoplasmic reticulum
ERK1/2: Extracellular signal-regulated kinase 1/2
ERN1: Endoplasmic reticulum to nucleus signaling 1
GRP78: Glucose regulatory protein 78
HFD: High-fat diet
HDL-C: High-density lipoprotein cholesterol
IL-1β: Interleukin-1 beta
IL-6: Interleukin-6
iNOS: Inducible nitric oxide synthase
LDL: Low-density lipoprotein
LDL-C: Low-density lipoprotein cholesterol
LPS: Lipopolysaccharide
MAPK: Mitogen-activated protein kinase
MCP-1: Monocyte chemoattractant protein-1
MMP: Matrix metalloproteinase
MTT: 3-(4,5-dimethylthiazol-2-yl)-2,5-diphenyltetrazolium bromide
NF-κB: Nuclear factor kappa B
NO: Nitric oxide
OSI: Oxidative stress index
ROS: Reactive oxygen species
T2DM: Type 2 diabetes mellitus
TAS: Total antioxidant status
TC: Total cholesterol
TP: Total peroxide
TG: Triglyceride
TNF-α: Tumor necrosis factor alpha
